# Tamoxifen induction of angiogenic factor expression in endometrium

**DOI:** 10.1038/sj.bjc.6600157

**Published:** 2002-03-04

**Authors:** S Hague, S Manek, M K Oehler, I Z MacKenzie, R Bicknell, M C P Rees

**Affiliations:** Nuffield Department of Obstetrics and Gynaecology, Women's Centre, John Radcliffe Hospital, Oxford OX3 9DU, UK; Molecular Angiogenesis Laboratory, Imperial Cancer Research Fund, Weatherall Institute of Molecular Medicine, John Radcliffe Hospital, Oxford OX 9DU, UK UK; Department of Cellular Pathology, University of Oxford, John Radcliffe Hospital, Oxford OX3 9DU, UK

**Keywords:** tamoxifen, angiogenesis, endometrium, vascular density, endothelial proliferation index

## Abstract

Tamoxifen is the current therapy of choice for patients with oestrogen receptor positive breast cancer, and it is currently under evaluation as a prophylactic for women at high risk of developing the disease. However, tamoxifen is also known to induce proliferative changes in the endometrium increasing the risk of developing endometrial hyperplasia, polyps and carcinoma. Angiogenesis is an intimate part of this process. For this reason, we have examined the expression of several well characterized angiogenic factors, namely, acidic and basic fibroblast growth factor, thymidine phosphorylase, vascular endothelial growth factor and adrenomedullin in both normal and tamoxifen exposed pre- and postmenopausal endometrium. Vascular density and endothelial proliferation index were also quantified. We found increased expression of acidic and basic fibroblast growth factor and adrenomedullin after treatment with tamoxifen mainly in premenopausal tissue. Vascular density was significantly increased in pre- but not post-menopausal endometrium (*P*=0.0018) following tamoxifen treatment. These results support the notion that angiogenesis is integral to the response to tamoxifen exposure, and is a potential target with which to block these side effects of tamoxifen.

*British Journal of Cancer* (2002) **86**, 761–767. DOI: 10.1038/sj/bjc/6600157 www.bjcancer.com

© 2002 Cancer Research UK

## 

The non-steriodal anti-oestrogen tamoxifen (TAM) is currently the long-term treatment of choice for selected patients with breast cancer, since large clinical trials have shown reduction in recurrence and overall survival benefit. Tamoxifen is currently being evaluated as a chemopreventative agent in healthy women at risk from the disease (Cuzick, 2000; Dalton and Kallab, 2001). One of the most significant complications of long-term TAM use is the development of endometrial cancer with up to a 7.5-fold increase in risk (Bergman *et al*, 2000; Carthew *et al*, 2000; Mourits *et al*, 2001). The increasing use of TAM, especially in healthy young subjects with no history of cancer, means that it is essential to improve our understanding of the mechanisms by which it exerts its adverse endometrial effects.

We have previously shown by differential PCR display that TAM, but not oestrogen induces expression of adrenomedullin (ADM) in endometrial isolates (Zhao *et al*, 1998). ADM stimulates angiogenesis in the chick chorioallantoic membrane assay (Zhao *et al*, 1998). ADM is also an autocrine growth factor for human endometrial endothelial cells and is clearly involved in endometrial angiogenesis (Nikitenko *et al*, 2000). ADM is pro-tumourigenic not solely due to its angiogenic activity but also because it enhances carcinoma cell survival under hypoxia by upregulation of Bcl2 (Oehler *et al*, 2001). ADM expression has been noted in a number of carcinomas (Hinson *et al*, 2000).

Other angiogenic factors which have been identified in endometrium, include vascular endothelial growth factor (VEGF) (Shifren *et al*, 1996; Zhang *et al*, 1998), thymidine phosphorylase (TP) (Zhang *et al*, 1997; Sivridis *et al*, 2000), basic fibroblast growth factor (bFGF) and acidic fibroblast growth factor (aFGF) (Ferriani *et al*, 1993). Vascular endothelial growth factor is now known to be a key regulator in neoplastic angiogenesis and its expression is upregulated in most human tumours (Ferrara and Davis-Smyth, 1997). Its expression is increased in human endometrial carcinoma (Doldi *et al*, 1996; Zhang *et al*, 1998).

The expression of thymidine phosphorylase is well documented in a number of carcinoma types (Brown and Bicknell, 1998) and has been previously documented in human endometrium (Zhang *et al*, 1997). It is also found in both simple and complex endometrial hyperplasias (Sivridis *et al*, 2000) but reports in human endometrial cancer are conflicting (Zhang *et al*, 1997; Fujiwaki *et al*, 1998; Sivridis *et al*, 1999). Increased expression of bFGF in endometrial hyperplasia has been documented (Gold *et al*, 1994). We are not aware of alteration of aFGF expression in endometrial carcinomas apart from carcinoma lines (Fujimoto *et al*, 1997).

The aim of the study was to determine whether TAM therapy changed the expression of a number of angiogenic factors in the endometrium in the absence of neoplastic change. Vascular density and endothelial proliferation index in normal and tamoxifen exposed endometrial samples were also documented.

## MATERIALS AND METHODS

### Preparation of tissue

Formalin fixed, wax embedded specimens of endometrium were obtained from hysterectomy samples selected from the archival files of the Histopathology Department of the John Radcliffe Hospital (Oxford, UK). Thirty normal premenopausal hysterectomy samples were obtained from women (aged 30–45) undergoing surgery for a subjective complaint of menorrhagia. No pelvic pathology was seen at operation. This was confirmed by a subsequent histological examination by an independent histopathologist. All patients had a history of regular 26–30 day menstrual cycles and had not used oral or intrauterine contraception, nor taken any hormones for at least 6 months prior to surgery. The stage of the menstrual cycle at which the tissue was obtained was determined from the patients menstrual history and endometrial histology (10=menstrual, 10=proliferative, 10=secretory) (Wells, 1996). In addition, 10 postmenopausal normal endometrial samples were obtained from women (aged 59–84) undergoing hysterectomy for uterovaginal prolapse. Again no pelvic pathology was evident and the women had not taken any hormones for at least 6 months. None of the controls had ever received tamoxifen.

Eight premenopausal hysterectomy samples (five proliferative, three secretory) and 13 postmenopausal hysterectomy samples derived from women who were treated with TAM (for between 3–5 years) were again obtained from the Histopathology Department of the John Radcliffe Hospital. The indication for hysterectomy was uterovaginal prolapse. The TAM exposed endometrial samples were neither malignant or hyperplastic nor did they contain polyps and were all the samples available collected over a 5 year time course. All tissues were collected in accordance with the Central Oxford Regional Ethics Committee (COREC C2519).

### Immunohistochemistry

Immunohistochemistry was essentially as previously described (Hague *et al*, 2000) briefly, all sections were dewaxed using Citroclear (HS Supplies, Aylesbury, UK), rehydrated sequentially in absolute, 95%, 70%, 20% ethanol, distilled water and finally rinsed in TBS (pH 7.6) prior to staining.

### aFGF and bFGF immunohistochemical staining

Immunohistochemical staining for aFGF was undertaken using the streptavidin-biotin peroxidase (ABC) method using the VECTASTAIN ABC kit for peroxidase (rabbit IgG) (Vector laboratories, Burlingame, USA) according to the manufacturer's protocol with rabbit anti-bovine aFGF polyclonal antibody (Sigma, Poole, Dorset, UK) at a dilution of 1 out of 200 in TBS/20% swine serum (SS). Sections were washed in TBS twice, and then developed with diaminobenzidine tetrahydrochloride (DAB) (Dako, Cambridgeshire, UK). Immunohistochemistry of bFGF used the same method with rabbit anti-bovine bFGF polyclonal antibody (Sigma, UK) diluted to 84 ng ml^−1^ in TBS.

### TP immunohistochemical staining

Immunohistochemical staining for TP was carried out using the streptavidin-biotin-alkaline phosphatase (ABC) method. Prior to application of the primary antibody, the sections were incubated with 20% normal rabbit serum (NRS) to block non-specific protein binding sites. Primary antibody, PGF44C (ICRF, UK) was added to the slides for 30 min. The slides were then washed twice in TBS and incubated for a further 30 min with biotinylated rabbit anti-mouse immunoglobulin at a 1 out of 50 dilution. The sections were washed again in TBS and incubated with streptavidin 1 out of 200 (Dako) for 30 min. Chromogen development was performed using a ‘New Fuchsin’ Substrate System (Dako) according to the manufacturers instructions by incubation for 5–10 min. Slides were counter stained with haematoxylin and mounted with Apathy's mounting medium

### VEGF immunohistochemical staining

Immunohistochemical staining for VEGF was carried out using the streptavidin-biotin-alkaline phosphatase (ABC) method, as previously described (Zhang *et al*, 1998). The ‘New Fuchsin’ Substrate System was used to visualise the sections. In the negative controls the primary antibodies were replaced with the same subtype of mouse immunoglubin (Sigma) at the same concentration.

### Adrenomedullin immunohistochemical staining

Immunohistochemistry was carried out as previously described (Zhao *et al*, 1998). Briefly, slides were incubated with 5% goat serum (Dako) to reduce non-specific background staining, followed by 1 out of 800 anti-adrenomedullin (Peninsula Laboratories, Liverpool, UK). A second biotinylated swine anti-rabbit antibody (Dako) at a dilution of 1 out of 400 was then applied for 30 min after which the slides were incubated with streptavidin 1 out of 200 (Dako) for 30 min, the final colour being developed with the ‘New Fuschin’ substrate system (Dako).

### Ki67/CD34 double staining

Immunohistochemical staining for CD34 was performed using the streptavidin-biotin-alkaline phosphatase (ABC) method. Throughout the protocol all antibody dilutions and washes were performed in TBS, with all incubations being performed at room temperature. Antigen retrieval was performed by means of pressure cooking in 1.6l 0.01 M sodium citrate buffer (pH 6.0) for 90 s, followed by a 30 min rinse in distilled water and 5 min in TBS. Prior to the application of the primary antibody the sections were blocked in 10% normal human serum. The sections were incubated at room temperature for 30 min with the primary CD34 antibody (Qbend 10, Novacastra, Newcastle upon Tyne, UK), diluted 1 : 25. Again the sections were rinsed for 2×5 min, followed by a 30 min incubation with the secondary biotinylated rabbit anti mouse IgG (Sigma), diluted 1 : 400. The sections were incubated with phosphate conjugated streptavidin at a dilution of 1 : 200 for 30 min. The final colour was developed with the New Fuschin substrate (Dako).

The Vectastain ABC kit was utilised for the staining of Ki67. Prior to staining, double stain enhancer (Zymed, San Francisco, CA, USA) was added to the sections; endogenous peroxidase activity was quenched by the application of 0.3% hydrogen peroxide, diluted in distilled water. The sections were washed, and incubated in horse serum, after which the primary Ki 67 (Bio Genex, San Ramon, CA, USA) antibody at a dilution of 1 out of 10 was added for 30 min. The sections were washed and incubated with the secondary biotinylated ABC antibody for 30 min, with the final colour being developed with DAB substrate (Sigma, UK).

### Scoring of immunohistochemical staining

The staining for aFGF, bFGF, TP, VEGF and ADM was scored on a scale of 1–3+, with 3+ indicating a strong positive result. The stroma, epithelium and endothelium were scored separately. Sections were examined by two reviewers (S Hague and S Manek). The final score, averaged across each section, was agreed upon by the two examiners.

### Determination of vascular density and endothelial cell proliferative indices

Vascular density was determined by Chalkley counting in three areas selected at random (Fox *et al*, 1995). Vessels were counted using a 25-point Chalkley eyepiece graticule at magnification ×250. The graticule was rotated in the eyepiece to where the maximal number of vessels were overlaid by graticule dots. Individual density was then obtained by taking the mean of three graticule counts.

The endothelial cell proliferative index was determined at ×400 magnification. The endothelial cell proliferative index was calculated as the percentage of all Ki-67 positively stained endothelial nuclei that also had concomitant positive cytoplasmic staining in CD34 positive cells

### Statistical analysis

Analysis of microvascular density, the endothelial cell proliferation index and angiogenic factor immunostaining, utilised the non-parametric Mann–Whitney *U*-test.

## RESULTS

### aFGF

**Figure 1:**
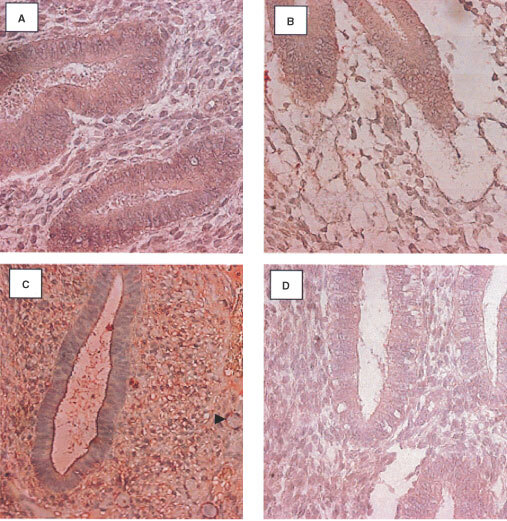
Localization of aFGF in the endometrium using a streptABC alkaline phosphatase method. Positive staining appears red. (**A**) Weak expression in the normal endometrium (early secretory), (**B**) weak aFGF expression in the epithelium and stroma of the post menopausal endometrium, (**C**) increased expression in the epithelium and stroma of the premenopausal tamoxifen samples (early proliferative). (**D**) weak expression in the epithelium and stroma of the post menopausal tamoxifen exposed endometrium (Magnification ×400) (Arrowhead indicates position of a blood vessel).

Negative or very weak aFGF expression was detected in normal endometrium, localising predominantly to the glandular epithelium, stroma and endothelium of the blood vessels (Figure 1A

). No change in the expression level of aFGF was observed through the menstrual cycle. In postmenopausal endometrial tissue, aFGF localised to the stroma, glandular epithelium and blood vessels in the control group (Figure 1B). The intensity of staining for aFGF expression in normal premenopausal and postmenopausal endometrium was compared (Table 1

**Table 1:**
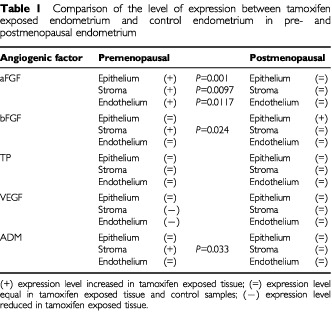
Comparison of the level of expression between tamoxifen exposed endometrium and control endometrium in pre- and postmenopausal endometrium

). Menopausal status did not significantly alter aFGF expression.

Expression of aFGF in the TAM exposed premenopausal endometrium was observed predominantly in the stroma and at a reduced level in the epithelium (Figure 1C). A comparison of the level of stain intensity between the premenopausal TAM exposed endometrium and the normal controls revealed that the level of aFGF staining was significantly higher in the glandular epithelium (*P*=0.001) stroma (*P*=0.0097), and in the blood vessels (*P*=0.0117) (Figure 1A compared to Figure 1C).

In the postmenopausal TAM exposed endometrial tissue, aFGF localized again to the stroma, glandular epithelium and blood vessels (Figure 1D). Comparison of the intensity of staining revealed no significant differences between the control and TAM exposed postmenopausal tissue (Figure 1B compared to Figure 1D). The staining intensity was higher in the premenopausal than in the postmenopausal TAM exposed tissue but did not achieve significance.

### bFGF

**Figure 2:**
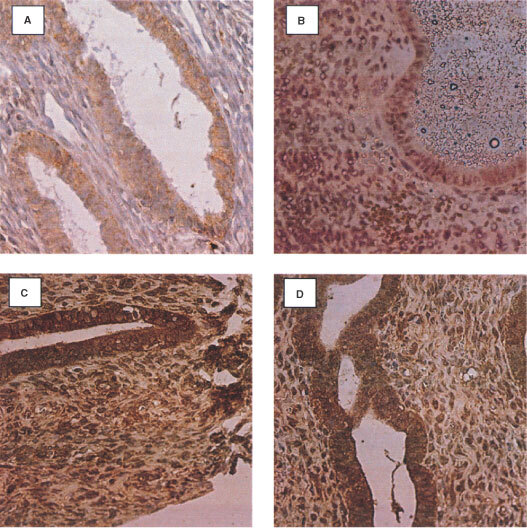
Localisation of bFGF in the endometrium using a streptABC peroxidase method. Positive staining appears in brown. (**A**) Expression in the normal endometrium (proliferative), (**B**) bFGF expression in the epithelium and stroma of the post menopausal endometrium, (**C**) increased expression in the epithelium and stroma of the premenopausal tamoxifen (proliferative) samples, (**D**) increased expression in the epithelium and stroma of the post menopausal tamoxifen exposed endometrium (Magnification ×400).

Unlike aFGF, expression of bFGF was observed in all of the normal endometrial sections examined, localising predominantly to the glandular epithelium and at a greatly reduced level, in the stroma and endothelium (Figure 2A

). Expression of bFGF increased in the proliferative phase and was reduced in the secretory phase of the menstrual cycle. In the postmenopausal control group, bFGF staining was observed in the epithelium, stroma and endothelium of the blood vessels (Figure 2B). The level was lower than that seen in the premenopausal control tissue. Statistical comparison, of bFGF intensity between pre and postmenopausal normal endometrium, showed that the level of expression was significantly higher in premenopausal glandular epithelial tissue (*P*=0.0005) and in the blood vessels (*P*=0.0142) but not in the stroma

Expression of bFGF in the endometrium of premenopausal TAM treated patients was evident in the stroma and epithelium, staining with approximately equal intensities, in contrast to the control group (Figure 2C). The intensity of staining remained constant during the cycle. The level of bFGF staining was significantly higher in the stroma (*P*=0.024) of the TAM exposed tissues, but not in the epithelium or endothelium (Figure 2A compared to Figure 2C).

Expression of bFGF was observed in the stroma, epithelium and blood vessels of the postmenopausal TAM exposed endometrium (Figure 2D). The level of expression in the epithelium of the TAM exposed tissue was significantly higher (*P*=0.0021) than that in the postmenopausal controls (Figure 2B compared to Figure 2D). Comparison of the staining intensities between the pre and postmenopausal TAM exposed endometrium, showed no significant difference.

### Thymidine phosphorylase (TP)

TP was detected in the control premenopausal endometrium throughout the menstrual cycle. Immunostaining was most intense in the glandular epithelium of the late secretory and menstrual phases. Staining of the endometrial stroma and endothelium was present at a lower level. TP expression was not detected. in the postmenopausal endometrium.

TP expression in the endometrium of premenopausal women exposed to TAM was evident in both the stroma and glandular epithelium. The degree of cyclical change in TP expression was not as apparent as in the normal premenopausal endometrium. No significant differences in the level of stain intensity for TP between the premenopausal TAM and control groups was found.

Several of the postmenopausal TAM exposed endometrial samples exhibited TP expression in the stroma and epithelium, however the majority of the samples did not. No statistical differences were observed in the expression level of TP in the postmenopausal controls and TAM exposed samples. While the level of staining intensity was higher in pre than in postmenopausal TAM exposed tissue, this did not achieve statistical significance.

### VEGF

VEGF was present in the endometrium of the control samples throughout the menstrual cycle and showed menstrual cycle-related expression in the specimens examined. It was principally detected in the glandular epithelium and at a reduced level in the stroma. As previously reported (Zhang *et al*, 1998), the highest level of staining was seen in the menstrual, early proliferative and late secretory phases of the cycle. Moderate staining of the small blood vessels was also observed with the endothelial component staining positive for the presence of VEGF. VEGF expression was primarily observed in the glandular epithelium of normal postmenopausal endometrium. VEGF expression was significantly increased in normal premenopausal tissue; epithelium (*P*<0.001), stroma (*P*=0.004), blood vessels (*P*=0.004).

Unlike the controls, VEGF expression was detected only in the epithelium of the TAM exposed endometrium. Its expression did not appear to alter with respect to the stage of the menstrual cycle and was not significantly increased. The TAM exposed postmenopausal tissues exhibited VEGF expression in the epithelium and occasionally in the stroma. There was no significant difference in expression levels between the postmenopausal controls and TAM exposed tissue.

When analysed according to menopausal status VEGF was significantly increased in pre- *vs* postmenopausal endometrium, with the premenopausal tissue exhibiting a significantly higher level of expression in the epithelium (*P*=0.033) but not in the stroma or the blood vessels.

### ADM

The stroma, epithelium, endothelium and macrophages of the normal premenopausal endometrium stained positive for ADM. The staining was present throughout the menstrual cycle, with no cycle specific change of expression. In the postmenopausal control samples, ADM expression was observed in the epithelium and stroma. Comparison of the expression levels between the control pre- and postmenopausal endometrium showed no statistically significant difference in the level of expression of ADM in the epithelium, stroma or blood vessel components of the endometrium.

Stroma, epithelium, endothelium and macrophages again stained positive for the presence of adrenomedullin, in the TAM exposed endometrium. In premenopausal tissues, TAM exposure significantly increased the level of staining in the stroma (*P*=0.00841) compared to the controls. No significant change was seen in the epithelium or the endothelium of blood vessels.

In postmenopausal TAM exposed endometrium, ADM expression was observed in the epithelium and stroma. Statistical analysis of the level of expression between the postmenopausal normal and TAM exposed tissue, revealed no significant difference in the levels of expression in the epithelium, stroma and blood vessels. Comparison of staining intensities in TAM exposed pre and postmenopausal endometrium, revealed a significant difference, with the premenopausal tissue exhibiting a significantly higher level of expression in the stroma (*P*=0.033).

### Vascular density and endothelial proliferation index

**Figure 3:**
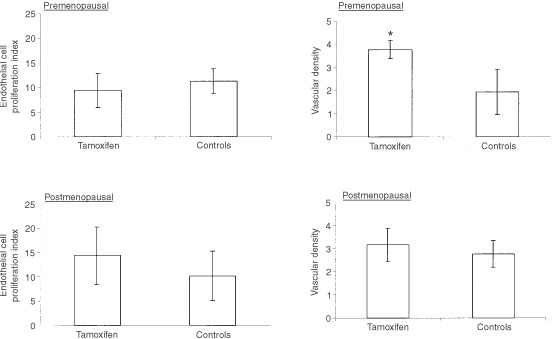
Comparison of the endothelial cell proliferation index and the vascular density between the premenopausal control (*n*=30) and tamoxifen (*n*=8) group and the postmenopausal control (*n*=10) and tamoxifen group (*n*=13) (*Indicates a significant *P* value of less than 0.005).

The vascular density of the endometrium was determined throughout the menstrual cycle by CD 34/Ki-67 double immunohistochemistry followed by Chalkley counting. The vascular density of the endometrium did not change significantly during the menstrual cycle (see Figure 3

). In premenopausal women, tamoxifen exposure significantly increased the vascular density (*P*=0.0018). CD 34/Ki-67 staining of the endometrium to quantify the endothelial proliferation index, revealed that endothelial proliferation did not significantly alter across the menstrual cycle. The endothelial proliferation index in the premenopausal TAM group was lower than that in the controls but the difference was not significant. The endothelial proliferation index in the post menopausal TAM group was higher than in the controls but again the difference was not significant.

## DISCUSSION

The side effects of TAM on the endometrium are thought to largely stem from the agonistic activity of the drug within this tissue. Adverse effects of TAM on the endometrium include hyperplasia, malignancy and uterine bleeding all of which involve angiogenesis.

We have examined the expression of a number of well characterized angiogenic factors in the endometrium of pre- and postmenopausal women receiving TAM therapy, namely aFGF, bFGF, TP, VEGF and ADM. Apart from ADM (Zhao *et al*, 1998), previous studies have mainly examined carcinoma lines or rodent uteri but not human tissues and not in relation to menopausal status. From the work described here, it appears TAM is capable of modulating the expression of these angiogenic factors, both in terms of the expression level and profile with aFGF, bFGF and ADM being upregulated especially in premenopausal tissue.

The effect of TAM exposure on aFGF has only been previously examined in the Ishikawa endometrial cancer cell line where it inhibits upregulation by oestradiol (Fujimoto *et al*, 1997). For bFGF, previous data are again limited to the Ishikawa endometrial cancer cell line where TAM inhibits its upregulation by oestradiol (Fujimoto *et al*, 1997).

Alteration in the pattern of expression by TAM was also observed with TP, with cyclical expression being replaced by a constant level in premenopausal tissues. Changes in the level of TP expression were not apparent when comparing control and TAM groups. Previous studies by ourselves have found that TP is not expressed in human endometrial adenocarcinomas (Zhang *et al*, 1997). However others have shown expression (Fujiwaki *et al*, 1998; Sivridis *et al*, 1999). Thus the role of TP in endometrial malignancy unclear.

Previous studies of the effect of TAM exposure on uterine VEGF expression have been mainly undertaken in rodents. The rat model showed an upregulation of VEGF expression by tamoxifen (Hyder *et al*, 1996).

ADM expression in response to TAM exposure appeared to be altered only in the premenopausal tissue studied, with expression being increased in the stroma. These findings concur with our previous results (Zhao *et al*, 1998).

Increased expression of a number of angiogenic factors has previously been documented in endometrial hyperplasias and carcinomas, including the overexpression of bFGF and VEGF in hyperplasia and adenocarcinoma (Gold *et al*, 1994; Doldi *et al*, 1996; Zhang *et al*, 1998). Adrenomedullin is increasingly being regarded as a significant factor in angiogenesis and tumour growth (Oehler *et al*, 2001). Indeed it has been demonstrated to be upregulated in a number of distinct tumour types (Hinson *et al*, 2000).

The upregulation of several angiogenic factors in non hyperplastic TAM exposed endometrium, supports the notion that it induces angiogenesis in the absence of neoplasia. The mechanism by which TAM may increase aFGF, bFGF and ADM expression is presently unknown. However the presence of an AP-1 site in the promoters of aFGF, bFGF and ADM (Ishimitsu *et al*, 1994; Erdos *et al*, 1995; Payson *et al*, 1998), provides a possible mechanism for the TAM induction of these factors, as it has been demonstrated that TAM is able to initiate gene transcription via an AP-1 site (Webb *et al*, 1995). None of these factors have oestrogen response elements.

Increased and malignant cell growth leads to a limited vascular supply and consequently to hypoxia. Both ADM and bFGF are known to be upregulated by hypoxia (Cormier-Regard *et al*, 1998; Nakayama *et al*, 1998; Le and Corry, 1999; Nguyen and Claycomb, 1999; Garayoa *et al*, 2000). TAM has been shown to induce hypoxia in MCF-7 xenografts (Evans *et al*, 1997). The induction of ADM and bFGF by hypoxia is under the control of the hypoxia inducible transcription factor-1 (Hif). Thus increased endothelial cell proliferation in response to TAM therapy, may be mediated by bFGF and ADM induced via Hif stabilization.

The vascular density and endothelial proliferative index were unaltered throughout the menstrual cycle in the endometrium. This concurs with previous studies (Rees *et al*, 1984; Hickey *et al*, 1996; Hague *et al*, 2000). Vascular density correlated with the proliferative index. The vessel density in the TAM exposed tissues was significantly greater in the premenopausal (*P*=0.0018) but not the postmenopausal samples. These findings concur with the upregulation of aFGF, bFGF and ADM in premenopausal tissue. Increased vascular density in premenopausal tamoxifen exposed endometrium is an important consideration for premenopausal women using TAM as a chemopreventative agent, especially since the trial results have been divergent (Fisher *et al*, 1998; Powles *et al*, 1998; Veronesi *et al*, 1998). Our results, suggest these women may be at an increased risk of abnormal endometrial angiogenesis. The endothelial proliferation index as determined by CD34/Ki-67 double immunohistochemistry failed to detect a significant difference in the endothelial proliferation rates both in the pre- and postmenopausal groups analyzed. The dichotomy between the increased vascular density and the lack of effect on endothelial proliferation may be explained by the fact that the women had been receiving TAM for over 1 year and that stimulation of endothelial growth may be an early event.

The absence of a direct effect of TAM on endothelial proliferation in the endometrium is in contrast to the effects of TAM on the breast where it has been found to be anti-angiogenic in responsive breast tumours (Gagliardi *et al*, 1996). This may be accounted for by TAM behaving as an agonist in the endometrium and an antagonist in responsive breast tumours.

In conclusion, the present study demonstrates the upregulation of several angiogenic factors mainly in premenopausal human endometrium in response to TAM therapy. Further, in premenopausal tissue examined, there was also an increase in vascular density. These results support the notion that TAM exposure stimulates endometrial angiogenesis in the absence of endometrial neoplastic change.
